# Applications of Antioxidants in Ameliorating Drugs and Xenobiotics Toxicity: Mechanistic Approach

**DOI:** 10.1155/2017/4565127

**Published:** 2017-08-29

**Authors:** Mohamed M. Abdel-Daim, Yasser M. Moustafa, Masakazu Umezawa, Kota V. Ramana, Elena Azzini

**Affiliations:** ^1^Pharmacology Department, Faculty of Veterinary Medicine, Suez Canal University, Ismailia 41522, Egypt; ^2^Department of Ophthalmology and Micro-Technology, Yokohama City University, Yokohama, Japan; ^3^Pharmacology Department, Faculty of Pharmacy, Suez Canal University, Ismailia, Egypt; ^4^Research Institute of Science and Technology, Tokyo University of Science, Tokyo, Japan; ^5^Department of Biochemistry and Molecular Biology, University of Texas Medical Branch, Galveston, TX 77555, USA; ^6^Council for Agricultural Research and Economics (CREA), Research Center for Food and Nutrition, Via Ardeatina 546, 00178 Rome, Italy

Biotransformation of drugs, xenobiotics, and environmental pollutants leads to the overproduction of free radicals in the body leading to lipid peroxidation, oxidative stress, and oxidative damage. The free radicals interact with various macromolecules, such as nucleic acids, proteins, and lipids and alter critical intracellular signaling pathways, responsible for the maintenance of cellular homeostasis. The free radicals, either directly or indirectly through the mediation of oxidative and inflammatory signals, disrupt the cellular equilibrium and cause mitogenesis, mutagenesis, genotoxicity, and cytotoxicity. The involvement of free radical-mediated damage in the pathophysiology of a variety of diseases including diabetes, hypertension, atherosclerosis, cancer, Parkinsonism, and Alzheimer's has been well established.

Currently, a plethora of phytochemical antioxidants have been developed in the therapeutic management to maintain normal homeostasis and for the prevention and treatment of many diseases and toxicities. The investigations on the molecular mechanisms through which antioxidants restrict free radical-mediated toxicity still remain the primary focus of many investigators for a better understanding of their biological activities as well as future drug development opportunities. The articles published in this special issue provide an in-depth understanding of antioxidants in the regulation of free radical-mediated disease pathologies.

An excellent review article by S. Shaban et al. discussed the cellular and molecular effects of antioxidant supplements on mitigation of oxidative stress, improvement of transplanted or cultured stem cell survival, potency, and differentiation. Further, they discussed how antioxidants enhance genomic stability, improve the stem cell adhesion to culture media, and enable researchers to manipulate cellular proliferation. This review nicely described the possible clinical applications of antioxidant-supplemented stem cells in improving neurogenesis in patients with neurodegenerative diseases and regeneration of infarcted myocardial tissue as well as in spermatogonial stem cell banking.

In this special issue, two research articles describe the benefit of antioxidants in protection against drugs and xenobiotics, using mouse models. The first study by M. Abdel-Daim et al. examined the beneficial role of citrus fruit-derived flavonoid (diosmin) on the oxidative damage induced by the antineoplastic and antirheumatic drug (methotrexate) in mice hepatic, renal, and cardiac tissues. Specifically, they have shown that diosmin prevents methotrexate-induced oxidative as well as inflammatory markers. Based on these results, they concluded that diosmin could be a promising agent to protect against methotrexate-induced toxicities in patients with cancer and autoimmune diseases. The second study by J. S. Ajarem et al. examined the protective effects of green tea (*Camellia sinensis*) on nicotine exposure-induced oxidative damage in mice leading to behavioral alterations in early childhood and young adulthood animals. This study demonstrates that green tea significantly improved the nicotine-induced abnormalities such as physical development, neuromotor maturation, and behavioral performance in newborn male and female mice.

Another interesting research article by X. Gong et al. highlighted a nephroprotective role of N-acetylcysteine amide (NACA) against contrast-induced nephropathy using rat model. They have shown that NACA ameliorates contrast-induced nephropathy by modulating the thioredoxin-1 and ASK1/p38 MAPK pathway, leading to inhibition of renal cell oxidative stress and apoptosis.

Further, two research articles describe the significance of antioxidants in broiler chicken. A. El-Far et al. demonstrated the improvement of growth performance, immunity, and antioxidant status in chickens, fed with a diet containing antioxidant-rich *Phoenix dactylifera* seeds, as compared to mannon-oligosaccharide-fed chicken. On the other hand, S. E. Abdo et al. reported the improvement of antioxidant gene expression as well as enhancement of chicken's resistance to heat stress in two commercial broiler strains (Ross 308 and Cobb 500), as a result of exposure to monochromatic blue light.

Another research article by J. Zhang et al. used NRK-52E cells to investigate whether the mitochondria-targeted antioxidant (MitoTEMPO) could protect against oxalate-mediated cell injury. Specifically, they found that MitoTEMPO regulates oxalate-induced cytotoxicity by modulating the oxidative stress and inhibiting the mitochondrial dysfunction. Thus, the authors suggested that MitoTEMPO could be a new candidate for the protection against oxalate-induced renal injury as well as urolithiasis.

Finally, M. El-Esawi et al. investigated the increased production of antioxidants, such as phenolics and flavonoids by hairy root cultures of *L. serriola*, transformed with *Agrobacterium rhizogenes* bearing the rolB gene. This study showed significant increases in the total phenolic and flavonoid contents as well as in the total reducing power of transgenic hairy lines, indicating the potential efficiency of hairy root induction platform in enhancing the antioxidant potential in *L. serriola*.

